# Prevalence of personal, work-related and patient-related burnout during the COVID-19 pandemic and its associated factors among healthcare workers in health clinics in the district of Manjung, Perak: A cross-sectional study

**DOI:** 10.51866/oa.597

**Published:** 2024-07-16

**Authors:** Yee Theng Lau, Hui Teng Chia, A Hadi Nadia, Marzuki Mohd Shaffari, Ismail Liliwati, Che Ismail Noor Syazana

**Affiliations:** 1 MD, MMed Fam Med, Klinik Kesihatan Lekir, Batu 8 Lekir, Sitiawan, Perak, Malaysia. Email: alysse_lyt@yahoo.com; 2 MD, MMed Fam Med, Klinik Kesihatan Ayer Tawar, Jalan Besar, Pekan Ayer Tawar, Ayer Tawar, Perak, Malaysia.; 3 MD, MMed Fam Med, Klinik Kesihatan Pulau Pangkor, Jalan Pasir Bogak, Pulau Pangkor, Perak, Malaysia.; 4 MD, MMed Fam Med, Klinik Kesihatan Sitiawan, Jalan Datuk Ahmad Yunus, Kg Serdang, Sitiawan, Perak, Malaysia.; 5 MD, MMed Fam Med, Klinik Kesihatan Pantai Remis, Jalan Tangki Air, Kg Sungai Batu, Pantai Remis, Perak, Malaysia.; 6 MD, MMed Fam Med, Klinik Kesihatan Beruas, Beruas, Perak, Malaysia.

**Keywords:** Burnout, Copenhagen Burnout Inventory (CBI), Healthcare workers, Social support

## Abstract

**Introduction:**

Burnout is a syndrome characterised by physical, emotional and mental exhaustion that results from a long period of involvement in an overwhelming work condition. It is prevalent among frontline workers. This study aimed to identify the prevalence of burnout among primary healthcare workers in the district of Manjung, Perak and determine the factors associated with burnout.

**Methods:**

This cross-sectional study was conducted among healthcare workers in seven health clinics located in the district from August to September 2022. The self-administered validated Malay version of the Copenhagen Burnout Inventory and the Malay version of the Multidimensional Scale of Perceived Social Support were used. These instruments consisted of 31 questions rated on a 5-point Likert scale. The scores were then summed up to determine the burnout level. Data were analysed using SPSS version 20. Simple logistic regression analysis was performed. Thereafter, multiple logistic regression analysis was conducted to determine the factors associated with burnout.

**Results:**

A total of 224 participants were included. Among them, 61.6% were nurses; 21.4%, doctors; and 17.0%, assistant medical officers. The prevalence of personal burnout was 31.3%; work-related burnout, 16.5%; and patient-related burnout, 5.4%. The factors associated with burnout were the highest educational level, financial difficulties and low perceived social support from friends and significant others.

**Conclusion:**

Healthcare workers in Manjung health clinics have a higher prevalence of personal burnout than work- and patient-related burnout. The findings of this study provide early insights and guidance for possible interventions.

## Introduction

Burnout is a syndrome due to stress from chronic workplace conditions that has not been managed successfully, characterised by physical and emotional exhaustion, increased mental distance from one’s job and decreased professional efficacy according to the 11 th Revision of the International Classification of Diseases.^1,2^ It can result in negativity in relation to one’s job, especially when dealing with patients, thus resulting in decreased professionalism and work efficacy.^3^ Burnout is prevalent among healthcare workers, especially frontliners.^3^ In the National Health and Morbidity Survey conducted in 2015 in Malaysia using the 12-item General Health Questionnaire, the prevalence of mental health problems among the general adult population was reported to be 29.2%.^4^ Burnout is found to be a risk factor for suicide and low quality of life, as affected individuals are prone to experiencing depression, anxiety and sleep difficulties.^5^ It is associated with sleep deprivation, family issues and feelings of being overwhelmed by assigned tasks.^5^ Therefore, measures aimed at reducing the stress levels of healthcare workers are needed to improve their general well-being and quality of life, consequently enhancing their efficiency at work.^5^

COVID-19 has emerged as a global health issue, which has resulted in an unprecedented demand for healthcare workers worldwide. It has caused a significant burden on the healthcare system.^6^

The disease has been associated with a remarkable rise in reported feelings of burnout. During the pandemic, healthcare workers faced issues such as insufficient personal protective equipment, risk of exposure to the disease, shortage of manpower, a lack of childcare, limited resources and lengthy working hours.^6-8^ Long working hours might cause physical exhaustion and increase the susceptibility to psychological distress. Consequently, these factors could worsen the quality of life and reduce the confidence and efficacy to perform well under extended working hours.^6,7^ Healthcare workers perceived that the quality of care and patient safety might be affected when their workload is increased and working hours are extended.^8^

A systematic review of studies conducted among healthcare workers in India demonstrated a prevalence of burnout ranging from 23% to 27%.^9^ In another study, the prevalence of burnout among healthcare providers in hospitals in Taiwan was reported to be 40.3%.^10^ A study in Indonesia reported a prevalence of burnout ranging from 70% to 88.3%.^11^ Some studies conducted on burnout among healthcare workers in Singapore showed burnout rates ranging from 40% to 60%, with most studies using the Maslach Burnout Inventory.^12-14^ In a study conducted in Malaysia, the prevalence of burnout was 51.3% among emergency healthcare workers.^15^ Another study performed in Sabah showed that the prevalence of personal, work-related and client-related burnout was 61.2%, 48.8% and 39.8%, respectively.^16^ There are many inventories used to assess burnout. In this study, the Copenhagen Burnout Inventory (CBI) was used to assess burnout among healthcare workers, as it is a valid and reliable instrument that can be easily applied among medical professionals compared to other inventories.^17^

The factors associated with burnout are job overload, frequent involvement with angry or difficult clients, stereotyped notion of underpaid work and lack of clear guidelines.^18^ Other factors significantly associated with burnout include younger age, underlying medical conditions, long working durations, direct dealing with patients with COVID-19, a lack of self-perceived social support at the workplace and inadequate childcare support.^19^ Working experience and working in different medical departments are also associated with burnout.^17^ This information is important to facilitate the planning of interventions to reduce the prevalence of burnout and further prevent progression to multiple implications related to personal and patient care, such as decreased professionalism, lack of empathy, increased medical errors, compromised patient safety and lack of teamwork. To date, there is a paucity of research on burnout among staff in health clinics during the pandemic, especially in Malaysia. Awareness, preventative strategies and early diagnoses can improve burnout among healthcare workers and thus improve their quality of life.

## Methods

This cross-sectional study was conducted from August to September 2022 among healthcare workers in seven health clinics in Manjung District. Manjung District is located in the south-western part of Perak State. It covers an area of 1113 km^2^ with a population of nearly 250,000 people and a population density of 200/km^2^. The district has few hundreds of primary care frontliners. Due to this large number, Manjung District was deemed an appropriate location for this study. Healthcare workers such as doctors, assistant medical officers and nurses were included as the study population because they were the frontliners who dealt with patients most of the time during the pandemic. In particular, doctors, assistant medical officers and nurses who were aged 18-60 years and were practising in health clinics in Manjung District were included. Conversely, healthcare workers who had pre-existing psychotic disorders, had a bipolar mood disorder, used illicit drugs, had alcohol dependence, were from other agencies doing attachment in the health clinics temporarily and were doing further studies (e.g. degree or post-basic studies) were excluded. The response rate was 100% among all categories of healthcare workers including doctors, assistant medical officers and nurses.

The formula for sample size calculation for prevalence studies,^15^ with a finite population size of 234 and precision of 0.025, was used. The sample size of 204 was calculated using the simple proportion formula for finite populations based on a prevalence of burnout among healthcare workers of 51.3%. Considering a 10% drop out rate, 224 participants were recruited. A total of 234 healthcare workers fulfilled the inclusion criteria. Among them, 224 were chosen using the simple random sampling function in the EpiCalc 2000 software (Brixton Health, www.brixtonhealth.com).

A data collection form that was used has 3 parts. It was pretested among five healthcare workers to assess its feasibility in the local setting. The results of the pretesting were not included in the final analysis. The questionnaire used had three parts, and permission was sought to use them. The first part of the questionnaire collected basic sociodemographic and clinical data including age, race, sex, religion, marital status, highest educational level, occupation, monthly household income, number of dependants, duration of service, distance between the house and workplace, medical conditions, working shift, working hours in a week, financial problems, problems with colleagues and family issues. The second part consisted of the self-administered validated Malay version of the CBI. The CBI is used to measure occupational burnout, with excellent psychometric properties, and is available in the public domain.^19^ It has three dimensions including personal (six items), work-related (seven items) and patient-related burnout (six items) (Cronbach’s alpha coefficient=0.83–0.87 for the three dimensions).^19^ Each question is scored on a 5-point Likert scale *(never, seldom, sometimes, often and always).* Each item is rated as follows: *always/to a very high degree* (score=100), *often/to a high degree* (score=75), *sometimes/somewhat* (score=50), s*eldom/to a low degree* (score=25) and *never/to a very low degree* (score=0). The score for the set of questions for each of the three burnout dimensions was added, and the average of the scores was calculated. An average score of ≥50% is considered to indicate burnout. The CBI was also validated in the pandemic context, with a Cronbach’s alpha coefficient of 0.94.^20^ The third part comprised the selfadministered validated Malay version of the Multidimensional Scale of Perceived Social Support (MSPSS-M). The MSPSS-M is used to assess social support from three sources: family, friends and significant others. It has 12 items, with four items for each source of support. Each item is scored on a 7-point Likert scale ranging from *very strongly disagree* (score=1) to *very strongly agree* (score=7). The total social support score is the sum of the scores for all 12 items. The greater the score, the higher the level of social support. Items 1, 2, 5 and 10 relate to support from significant others; items 3, 4, 8 and 11, from family; and items 6, 7, 9 and 12, from friends. The item scores are summed and then divided by 4 to calculate the subscale scores. The total scale score is calculated by adding all 12 item scores and then dividing the sum by 12. A total score of 1–2.9 indicates low support; 3–5, moderate support; and 5.1–7, high support. The instrument showed good internal consistency (Cronbach’s alpha coefficient=0.89), parallel-form reliability (0.94) and test–retest reliability (0.77) (Spearman’s rho, P<0.01).^21^ All participations were voluntary, and written consent was obtained before questionnaire completion.

Permission to conduct this study was acquired from the NMRR [approval number: NMRR 22-01231-B0J (IIR)]. Data were analysed using SPSS version 20. Descriptive analysis was performed using frequencies and percentages. Age, monthly household income, duration of service, number of dependants, distance of the workplace from the house and total working hours per week were presented as medians, as these data were not normally distributed. Simple logistic regression was used to select variables for further analysis. All variables with P-values of <0.25 and clinically significant variables were included in multiple logistic regression. This P-value was set higher than the level of significance to allow for more important variables to be included in the model. The independent factors associated with burnout were identified via multiple logistic regression. P-values less than 0.05 were considered statistically significant.^16^

## Results

The median age of the participants was 37 years (IQR=10.0). Majority of the participants were Malays (87.5%), followed by Indians (6.7%) and Chinese (4.0%). There were more female participants (80.8%) than male participants (19.2%). Most participants held a diploma (47.8%). Approximately 61.6% of the participants were nurses. The sociodemographic and clinical characteristics of the participants are detailed in Table 1.

**Table 1:** Sociodemographic and clinical characteristics of the participants.

Variables	n (%)	Median (IQR)
**Age**		37 (10.0)
**Sex**		
Female	43 (19.2)	
Male	181 (80.8)	
**Race**		
Malay	196 (87.5)	
Chinese	9 (4.0)	
Indian	15 (6.7)	
Others	4 (1.8)	
**Religion**		
Islam	198 (88.4)	
Buddha	3 (1.3)	
Hindu	14 (6.3)	
Others	9 (4.0)	
**Marital status**		
Single	15 (6.7)	
Married	203 (90.6)	
Divorced	4 (1.8)	
Widow/widower	2 (0.9)	
**Highest educational level**		
Secondary	64 (28.6)	
Diploma	107 (47.8)	
Degree and above	53 (23.7)	
**Occupation**		
Doctor	48 (21.4)	
Medical assistant	38 (17.0)	
Staff nurse/community nurse	138 (61.6)	
**Monthly household income (RM)**		5000(4000)
**Duration of service (month)**		59.5 (91)
**Number of dependants**		3 (2)
**Distance from the house to the workplace (km)**		10 (16.48)
**Working shift**		
No	195 (87.1)	
Yes	29 (12.9)	
**Total working hours in a week**		45 (0)
**Underlying chronic medical illnesses**		
No	183 (81.7)	
Yes	41 (18.3)	
**Financial problem**		
No	201 (89.7)	
Yes	23 (10.3)	
**Colleague-related problem**		
No	214 (95.5)	
Yes	10 (4.5)	
**Family problem**		
No	213 (95.1)	
Yes	11 (4.9)	

The prevalence of personal burnout among the participants was 31.3%; work-related burnout, 16.5%; and patient-related burnout, 5.4%. The assistant medical officers and staff nurses had 3.93 times higher odds of developing personal burnout than the community nurses [95% confidence interval (CI)=1.55, 10.01; P=0.004]. The doctors had 8.36 times higher odds of experiencing personal burnout than the community nurses (95% CI=3.05, 22.95; P=0.000). The participants with financial problems had 5.93 times and 4.96 times higher odds of developing personal burnout (95% CI=2.13, 16.52; P=0.001) and work-related burnout (95% CI=1.98, 12.42; P=0.001) than those without, respectively. The participants with low perceived social support from significant others had 14.91 times higher odds of experiencing personal burnout than those with high perceived social support from significant others (95% CI=1.42, 157.03; P=0.024). The participants with low perceived social support from friends had 49.50 times higher odds of developing patient-related burnout than those with high perceived social support from friends (95% CI=4.45, 550.32; P=0.010) (Table 2).

**Table 2:** Multiple logistic regression analysis of the factors associated with burnout among the participants.

Variables	Multivariate analysis				
	aOR	95% CI	Wald statistic	df	P-value
**Highest educational level** Secondary Diploma Degree and above	1.00 3.93 8.36	(1.55,10.01) (3.05,22.95)	8.257 16.984	2 1 1	0.000 0.004 0.000
**Financial problem**					0.000
No	1.00				
Yes	5.93	(2.13,16.52)	11.562	1	0.001
**Suppoit from significant others**					0.044
High	1.00			2	
Moderate	1.55	(0.81,2.98)	1.732	1	0.188
Low	14.91	(1.42, 157.03)	5.059	1	0.024
**Work-related burnout Financial problem**					0.000
No					
Yes	4.96	(1.98,12.42)	11.664	1	0.001
**Patient-related burnout Suppoit from friends**					0.013
High	1.00			2	
Moderate	3.57	(0.74,17.20)	2.512	1	0.113
Low	49.50	(4.45, 550.32)	10.082	1	0.010

aOR, adjusted odds ratio; CI, confidence interval

## Discussion

The overall prevalence of personal burnout among the healthcare workers in health clinics in Manjung District was 31.3%; work-related burnout, 16.5%; and patient-related burnout, 5.4%. This finding is consistent with that of a study conducted in Japan, wherein the overall prevalence of burnout was 31.4%.^22^ However, the study was performed in a tertiary hospital. Conversely, the prevalence noted in the present study is much lower than that reported in a study on Malaysian healthcare workers: 53.8%, 39.1% and 17.4% for personal, work-related and patient-related burnout, respectively.^18^ This could be because this study was conducted when Malaysia was in its third month under a movement control order during the initial phase of the COVID-19 pandemic; in comparison, our study was performed among doctors, assistant medical officers and nurses in health clinics in a district during the end of the pandemic, when the number of cases had significantly dropped and movement control measures were eased. Even before the pandemic, high levels of personal, work-related and patient-related burnout were also found in a study conducted among doctors in Sabah, with rates of 57.1%, 48.8% and 30.4%, respectively.^23^

The current study showed that the healthcare workers who held a diploma had higher odds of experiencing personal burnout than those who completed secondary school only. The healthcare workers who held a diploma were the assistant medical officers and staff nurses. The participants who held a degree and above had 8.4 times higher odds of developing personal burnout than those who completed secondary school only. Similarly, Font et al. showed that doctors faced higher levels of burnout than nurses in Spanish oncology units.^24^ Another study found that workers employed in government service had relatively lesser job flexibility and autonomy than those employed in the private sector.^25^ Healthcare workers who held a diploma encountered higher job demands surpassing resources.^25^ Increased workload among healthcare workers including multitasking during the pandemic has resulted in higher risks of burnout by depleting the capacity of workers to meet their job demands.^26^ Further, negative emotions of patients and colleagues have triggered similar emotions in healthcare workers with a diploma, making them more susceptible to burnout.^27^

We also found that personal and work-related burnout were associated with financial difficulties. This is consistent with the findings of a study among radiology trainees, wherein financial constraint was a risk factor for burnout.^26^ Similarly, financial difficulties were found to be associated with high burnout levels among a female general population in another study.^25^ These findings indicate that further attention is needed to assess and help healthcare workers with financial constraints through, for example, training in financial planning via courses.^28^ If financial problems are solved, the risk of personal and work-related burnout can be reduced.

Social support from friends and significant others plays an important role in reducing the risk of burnout by acting as a buffer. Providing support can be an essential psychological approach with better efficacy than pharmacological therapy. A lack of social support is related to increased burnout levels. Social support from friends is associated with lower work-related burnout levels.^28^ This suggests the importance of creating policies that do not mandate separating frontline healthcare workers from their friends or significant others. Healthcare workers can easily seek psychological support from their family members to relieve stress from work instead of feeling isolated or helpless.^26^

According to a systematic review, decreased social support is a vital risk factor for the development of psychological issues among healthcare workers, especially during disasters.^29^ Support from families, friends, colleagues and healthcare organisations can provide healthcare workers the opportunity to alleviate negative feelings and emotions, which can then reduce the risk of burnout syndrome.^29,30^ Individuals can cope with difficulties by receiving support from their significant others.^28^ Several studies have shown that strong social support during the COVID-19 pandemic can reduce feelings of isolation and, consequently, the risk of burnout among healthcare workers.^28,30^

A limitation of this study is that the findings might not be generalised to other healthcare workers, as their job scopes differ. Nonetheless, the study provides an estimation of the prevalence of burnout among healthcare workers in a similar workplace setting in Malaysia. Another limitation is the possibility of reporting bias, as the burnout data were obtained using a self-administered instrument. Despite the limitations, the findings provide insights into burnout among healthcare workers in the country.

## Conclusion

Burnout is prevalent among healthcare workers in Manjung District. Several measures could be implemented to reduce the mental health impact of burnout on healthcare workers, including mental health screening, immediate access to mental healthcare services, early intervention and establishment of support groups. Further studies can be conducted in the country to better identify the issues and contributing factors of burnout.

## Data sharing statement

Raw data are available only upon request.

**How does this paper make a difference in general practice?**

- This study examined the determinants of burnout among healthcare workers in primary care in Manjung District.
- The findings could assist public healthcare authorities in designing a targeted promotional and intervention programme to reduce the risk of burnout among healthcare workers in primary care.
